# Disclosure of Financial Conflicts of Interests in Interventions to Improve Child Psychosocial Health: A Cross-Sectional Study

**DOI:** 10.1371/journal.pone.0142803

**Published:** 2015-11-25

**Authors:** Manuel Eisner, David K. Humphreys, Philip Wilson, Frances Gardner

**Affiliations:** 1 Institute of Criminology, University of Cambridge, Cambridge, United Kingdom; 2 Centre for Evidence-Based Intervention, Department of Social Policy and Intervention, University of Oxford, Oxford, United Kingdom; 3 Centre for Rural Health, University Of Aberdeen, Centre for Health Science, Inverness, United Kingdom; McGill University, CANADA

## Abstract

Academic journals increasingly request a full disclosure of financial conflict of interest (CoI). The Committee for Publication Ethics provides editors with guidance about the course of action in the case of suspected non-disclosure. No prior study has examined the extent to which journal articles on psychosocial interventions disclose CoI, and how journal editors process requests to examine suspected undisclosed CoI. Four internationally disseminated psychosocial interventions were examined. 136 articles related to an intervention, co-authored by intervention developers and published in health sciences journals were retrieved as requiring a CoI statement. Two editors refused consent to be included in the study. COI disclosures and editor responses were coded for 134 articles. Overall, 92/134 (71%) of all articles were found to have absent, incomplete or partly misleading CoI disclosures. Disclosure rates for the four programs varied significantly between 11% and 73%. Journal editors were contacted about 92 published articles with no CoI disclosure or a disclosure that was considered problematic. In 65/92 (71%) of all cases the editors published an ‘erratum’ or ‘corrigendum’. In 16 of these cases the journal had mishandled a submitted disclosure. The most frequent reason for non-publication of an erratum was that the journal had no disclosure policy at the time of the publication (16 cases). Consumers of research on psychosocial interventions published in peer-reviewed journals cannot currently assume that CoI disclosures are adequate and complete. More efforts are needed to achieve transparency.

## Introduction

Over the past decade clinicians and researchers have become progressively sensitized to the potential for research to be biased due to financial conflicts of interest (CoI).[1–3] Much recent concern relates to situations where pharmaceutical companies sponsor research about the effectiveness of drugs.[4, 5] CoI associated with psychosocial interventions has received less attention. However, health services in many countries increasingly rely on commercially disseminated psychosocial interventions that claim support by research evidence.[6] They play an increasing role in officially approved selections of evidence-based interventions that address conduct problems, substance use, or mental health issues.[7] Financial arrangements for such interventions vary, but implementation often benefits the program developers. Mechanisms include the ownership of companies that distribute the intervention, the receipt of royalties and consulting fees, and the sponsorship of research by the disseminating organization.

Guidance by the Committee of Publication Ethics (COPE) for publishers and journal editors is an important basis for improving research transparency and integrity. The Code of Conduct published in 2003 specified that editors of journals which are members of COPE should have systems in place that manage authors’ conflicts of interests. Hence author guidance in most journals across the health sciences now requires a full disclosure of potential conflicts of interests. Also, COPE provides editors with guidance about the appropriate course of action when a reader reports a suspected undisclosed CoI in a published article.[8]

This article examines papers published in peer-reviewed journals and co-authored by the developer of one of four major internationally disseminated psychosocial interventions addressing parenting: The Triple P (*Positive Parenting Program)* program developed by Matthew Sanders,[9] the early home-visiting program, *Nurse-Family Partnership* by David Olds,[10] the parenting and social skills program *Incredible Years* by Carolyn Webster Stratton,[11] and the therapeutic intervention primarily for youth offenders *Multi-Systemic Therapy* by Scott Henggeler.[12]

These interventions were selected for three reasons: They went through a similar product development cycle from initial trials in the 1980s to global dissemination in the last 10 years; they are professionally disseminated in multiple service settings (e.g. clinical, public health, youth justice, early childcare) in multiple countries; and in all four cases the developers have some financial conflict of interest.

Triple P is a system of standardized parenting support interventions based on social learning and cognitive-behavioral principles.[9] It is one of the most widely evaluated parenting programmes worldwide. The evidence-base for Triple P is controversial. A meta-analysis of 101 studies conducted by authors with a financial conflict of interests concluded that Triple P has positive effects on a broad range of child, parent, and family outcomes.[9] However, an independent systematic review found no convincing evidence that Triple P has positive effects across the whole population or in the long run.[6, 13] Triple P began commercial operations in 1996. In 2001 the University of Queensland granted the worldwide dissemination license to the private company Triple P International (TPI).[14] About seven million copies of group-based standard Triple P have been sold in 18 languages across 25 countries, and over 62,000 providers have been licensed.[14] The licensing contract between the University of Queensland and TPI includes the transfer of royalties from the sale of Triple P to UniQuest, which then distributes them to three groups of beneficiaries: the University of Queensland; the Parenting and Family Support Centre; and the authors of Triple P.[15] This includes Matthew Sanders, the creator of Triple P, as the primary recipient. Other authors have contributed specialized versions of Triple P and are also entitled to royalties. The primary program developer also receives consulting fees, and his research activities benefit from the sale of Triple P products as one third of the royalties are paid to his Parenting and Family Support Centre.

Incredible Years is an evidence-based system of interlocking programs that aims to promote a healthy social and emotional development, to support problem solving abilities, and to reduce problem behaviors among children aged 0 to 12.[16] It comprises parent training programs, child programs, and group management programs for teachers and childcare providers. A systematic review of 50 studies concluded that Incredible Years was effective in improving child behavior in a diverse range of families.[17] Since it was first introduced in 1987, Incredible Years has diversified the age range it serves. Over 30,000 staff have been trained in IY worldwide, and the program is currently available in 24 countries.[18] It is disseminated through a private company, Incredible Years, Inc. The CoI arrangements are unusual in that Carolyn Webster-Stratton, the initiator and main program developer of IY, has voluntarily distanced herself from activities that could potentially bias the research findings throughout the period of her academic affiliation with the University of Washington. The arrangement is described as follows in the standard CoI disclosure: “Dr. Webster-Stratton has disseminated these treatments and stands to gain from favorable reports. Because of this, she has voluntarily agreed to distance herself from certain critical research activities, including recruitment, consenting, primary data handling, and data analysis. The University of Washington has approved these arrangements.“[19]

The Nurse Family Partnership (NFP, known in the USA as the Family-Nurse Partnership) is an intensive evidence-based home visitation program for low-income first-time mothers. The program begins during pregnancy and continues for the first two years following birth. It aims to help mothers improve their prenatal health, supports parents’ early care of their children, and assists mothers with subsequent planning of education and work. NFP was initially developed by David Olds in the 1970s.[20] Studies conducted in the United States suggest positive effects of the programme on parental care, a reduction in child maltreatment, improvements in the maternal life-course.[21, 22] A recent replication study in England found no additional benefit over services as usual on the primary outcomes.[23] The Nurse Family Partnership has grown enormously since the initial research in the 1970s and 1980s. It is currently available in over 40 states of the USA and is implemented in six countries internationally. In 2003 the Nurse-Family Partnership National Service Office, a non-profit organization, was established. It facilitates replication of the NFP program across the US and provides agencies with support in nursing education, program quality assurance, marketing, and public policy. The developer does not receive personal remuneration from the licensing of the program. However, the University of Colorado Denver receives funds from the licensing and some of these funds go to the Prevention Research Center for Family and Child Health (PRC), directed by Dr. Olds. The mission of this research centre is to conduct research on the NFP. Also, university salaries of academic staff, including that of the salary of the program developer, are partly funded through the contract between the University of Colorado and the Nurse-Family Partnership National Service Office.[24]

Multisystemic Therapy (MST) is an intensive family-focused and community-based treatment program for chronic and violent delinquent adolescents. It was originally developed by Scott Henggeler in the late 1980s and adopts a socio-ecological approach.[25] Various trials suggest that Multisystemic Therapy is effective for decreasing delinquency and other externalizing behaviors including substance use, and in reducing the likelihood of out-of-home placement.[26] In the past 15 years MST has been increasingly disseminated internationally. MST is currently available in 34 US states, 15 countries worldwide, with 23,000 young people treated each year. In 1996 MST Services was founded as a private for-profit company that oversees the worldwide dissemination of the program and provides supervision to clinicians. MST Services has an exclusive license from the Medical University of South Carolina for intellectual property. The main program developer is a member and stockholder of MST Services.[27]

No prior study has examined the extent to which journal articles on commercially disseminated psychosocial interventions and co-authored by programme developers fully disclose conflicts of interests, and how journals in the health and psychosocial disciplines process requests to examine suspected undisclosed CoI in a published article. The present article addresses this issue by examining all articles relating to four major psychosocial interventions published between 2008 and 2014. It aims to provide empirical findings about the extent to which COI disclosures in the field of psychosocial interventions are complete, and the ways in which editors respond to requests to examine potentially missing or incomplete COI disclosures.

## Methods

The present study entailed two stages. The first stage relied on data in the public domain. It entailed the retrieval of all relevant articles in peer-reviewed journal articles co-authored by the programme developer and the coding of COI disclosures. The second stage entailed contacting editors in all situations where a COI disclosure had been coded as absent, potentially wrong or incomplete, and the coding of the editors’ responses.

All articles co-authored by the primary program developers and published in English between January 2008 and July 2014 were examined. 2008 was chosen as the start year because the first COPE guidance for responding to undisclosed CoI was published in 2006.[8] We assumed that by 2008 these rules were widely in force. COPE guidance rather than guidance by other professional bodies such as the International Committee of Medical Journal Editors (ICMJE) was used because we anticipated that much research on psychosocial interventions would be published in the fields of prevention science, psychology, nursing, education sciences, criminology and public health—areas where journals are probably most likely to be members of COPE.

Google Scholar, PubMed and web-based CVs were used to find all publications co-authored by the four developers, using the names of the authors as the search criterion. Two research assistants were given written instructions about data retrieval and initial coding. They read all articles that could be located, and recorded information relevant for determining a potential CoI on a Microsoft Excel coding sheet. Where present, CoI disclosures were recorded. A total of 176 publications were retrieved (S1 Data). In a next step we determined whether a publication required a CoI statement: Publications were coded as potentially requiring a CoI statement if they relate to the registration, design, or findings of pertinent trials; if they were overviews of the empirical or theoretical bases of the intervention; or if they discussed research on provider training and implementation. Research assistants conducted the initial coding. All coding was reviewed by the first author of this study. The coding of doubtful cases was resolved through joint deliberation. In a few cases it was determined that it was unclear whether the article related to a program or a program component, and a CoI disclosure was therefore appropriate. These cases were coded as potentially requiring a CoI disclosure at this stage, and were hence included for further clarification in the second stage of the analysis.

We then coded whether publications adequately reported a CoI. The operational definitions for the classification used in this study are reported in S1 Appendix. Subsequently we refer to code labels used there. Articles were coded as disclosing a conflict of interest and not requiring a contact with the editor if they provided information about whether income from the program dissemination contributes to research by the developer, information about royalties or consulting fees paid to the developer, or information about developer ownership of the disseminating company (code C). For all publications that required further clarification we distinguished three categories: Papers with no CoI disclosure statement (code D1), articles that actively reported “no financial conflict” without any qualification (code D2), and articles that had either unclear CoI statements or contradictory information (code D3). For example, some disclosures stated “the authors report no financial conflict”, but went on to report relevant financial arrangements in the disclosure text.

In the second stage of the study we contacted journal editors about missing or incomplete CoI statements, asked for clarification about the journal’s CoI policy, and requested that publication of an erratum be considered in line with COPE guidance.

Between July 2014 and July 2015 the first three authors of this study contacted the editors of journals where an intervention-related paper had been published without a CoI disclosure. The email listed the articles deemed problematic, described the nature of the financial CoI of the program developer, and requested clarification of the situation, including a ‘corrigendum’ or ‘erratum’ if appropriate. It also referred to the journal’s CoI policy if such a policy was specified in the author instructions.

Progress was monitored by means of a Microsoft Excel tracking sheet, and the final outcome was coded. Codes were developed to reflect the main types of outcomes. Four reasons for non-publication of an erratum were distinguished, namely a) the editor reported that the journal did not have a CoI disclosure policy when the manuscript was processed (code E1); b) the editor examined the issue with the authors and reported that we had erroneously assumed the presence of a CoI, for example because the examined treatment was not part of the disseminated program (Code E2), c) the editors were unwilling or unable to examine the issue, for example because the relevant documents were no longer available or they requested that we contact the authors directly (code E3), d) we did not obtain a response after at least three reminders (code E4). Reminders were generally sent out 2–4 weeks after the initial contact or a preliminary response, but took into consideration ‘out of office’ notices or announcements by editors of when we could expect a response. We waited for up to 30 days for an initial response by the editor.

Two reasons for the eventual publication of an erratum or corrigendum were distinguished: The first refers to cases where the editors discovered an error in the original handling of the manuscript, which then led to the erroneous non-publication of a submitted CoI disclosure (code F1). The second group refers to cases where the editor contacted the authors and a new or revised CoI disclosure was submitted for publication as an erratum (code F2).

### Variables associated with CoI disclosure

We also examined five variables that may be associated with the likelihood that a CoI is disclosed: First, to examine whether there was a trend towards more CoI disclosures since the introduction of COPE guidance two periods were distinguished, namely 2008–11 and 2012–14. Second, we examined whether the affiliation of a journal to the Committee of Publication Ethics COPE influenced CoI reporting. Coding was based on the most current list of COPE members and may not necessarily have been correct at the time of the publication of the article. Journals that are not members of COPE may be members of other associations such as the American Psychological Association that have their own ethical standards for editors in connection with CoI disclosure. Third, because the first authors often are responsible for submitting all details related to a publication, a dummy variable was formed to measure whether the program developer was the first author or not. Fourth, we coded whether journals had a published and compulsory CoI disclosure policy for authors at the time the article was published. Published author instructions were screened to determine whether the journal requested the disclosure of CoI. Journals were coded as not having a published disclosure policy if no such request was included in the author instructions or if the journal editor informed us that no such policy had been in place when the article had been published. Finally, we coded whether the subject area of the journal was predominantly in psychiatry, pediatrics and the medical sciences or not. The reason was that the medical field probably began to address the issue of undisclosed CoI and potential research bias associated with CoI earlier than psychology and the prevention sciences.

### Ethics statement

The first stage of the study used data that are in the public domain. For the second stage the journal editors were contacted in their professional role as representatives of the journal. Authors of this article contacted the journal editorial offices in their role as users of academic research, requesting that a missing CoI disclosure be examined in line with COPE guidelines. At this stage the authors did not disclose to the journal editors that their responses could subsequently be used for research purposes. This approach is based on the argument that journal editors respond to reader requests in a public role, providing a public service to the research communities they serve. In such situations researchers can legitimately put themselves in the position of service users, ‘mystery shoppers’, or academic citizens.[28] All journal editors were debriefed after the completion of the study, including full information about the study purpose and all findings presented in this article. The debriefing included an invitation to comment on the findings and a request for consent to the inclusion of the journal responses in the study. Two journal editors representing two publications requested that their decisions were not included in the study. The procedure was approved by the ethics committee of the Institute of Criminology of the University of Cambridge.

## Results

Table 1 shows summary statistics for the 176 articles published in 90 different journals. The most frequent subject areas of the journals were pediatrics, psychiatry, clinical psychology, counseling and family studies, prevention science, and public health. Initial screening showed that 40 (23.9%) of the publications were not related to the psychosocial interventions under examination or could not be found, for example because the journal had ceased to exist (Table 1). The remaining 136 publications (77.1%) were assessed as potentially intervention-related and hence requiring a CoI statement (Table 1). Two editors wished not to be included in the study. The sample available for analysis was therefore 134 articles in 73 journals. Of these 51% (n = 68) had been published in 2012–2014: 67% (n = 90) of articles had been published in a journal that was a COPE member at the time of the publication; the program developer was the first author in 32% (n = 44) of the articles; the journal had a published COI disclosure policy in 32% of the articles and 17% (n = 23) of the articles had been published in journals that are predominantly related to the medical sciences.

**Table 1:** Summary Statistics for retrieved articles, program related articles, and criteria examined for association with COI disclosure.

	n
Retrieved articles	
Articles co-authored by program developer, Jan 2008–July 2014	176
Different journals	90
Of which journals retracted from study (no informed consent)	2
Articles available for analysis	174
of which not related to program	40
Sample included in study	
Program-related articles	134
Different Journals	73
Criteria examined for association with COI disclosure	
Published Jan 2012–Jul 2014	68
Journal is a COPE member “YES”	90
Program developer is the first author	44
Journal has published COI disclosure policy	44
Journal discipline psychiatry, pediatrics and medical science	23

Table 2 shows the main results for the relevant articles. The majority of publications, namely 59%, related to Triple P. Ten percent related to NFP, 19% to MST, and 12% to IY. Editors of 58 journals were contacted in connection with n = 92 articles. The most frequent subject areas of the journals were pediatrics, psychiatry, clinical psychology, counseling and family studies, prevention science, and public health.

**Table 2:** Publications in peer-reviewed journals with and without CoI disclosures, four internationally disseminated psychosocial interventions, Jan 2008–July 2014.

Row^1^	Characteristic	Triple P	NFP	MST	IY	Total
**B**	**Included in analysis**	**79**	**14**	**25**	**16**	**134**
**C**	**COI fully disclosed, editor not contacted**	**8**	**8**	**16**	**10**	**42**
**D**	**Editor contacted**	**71**	**6**	**9**	**6**	**92**
D1	CoI disclosure missing	60	4	9	6	79
D2	"No conflict of interest" statement	4	1	0	0	5
D3	Ambiguous or incomplete disclosure	7	1	0	0	8
**E**	**No erratum/corrigendum published**	**13**	**5**	**6**	**2**	**26**
E1	No disclosure policy	11	3	1	1	16
E2	Not program paper–journal/author response	0	0	3	0	3
E3	CoI deemed sufficient	1	0	0	0	1
E4	Unable/unwilling to examine	1	2	1	1	5
E5	No final response	1	0	1	0	2
**F**	**Erratum/corrigendum announced**	**57**	**1**	**3**	**4**	**65**
F1	Journal mishandling	14	0	2	0	16
F2	Authors submit corrected or new CoI	43	1	1	4	49
	**Rates**					
	Disclosure rate^2^	11%	57%	73%	63%	33%
	Errata rate^3^	80%	17%	33%	67%	71%

Notes

^1^ See S1 Appendix for coding scheme and operational definitions.

^2^ Calculated as (C+E3)/(B-E2).

^3^ Calculated as F/D.

Overall, 42 publications were assessed as having adequate CoI disclosures in the initial stage of the study. Subsequent responses by editors showed that three publications attributed to MST were unrelated to the psychosocial intervention and were therefore removed from the relevant calculations (code E4). One CoI disclosure related to Triple P that we had considered incomplete was found to have been adequate by the group of editors. It was therefore reclassified for the analyses. Taking these changes into account the overall rate of adequate CoI disclosures was 32%. There were significant differences in disclosure rates between programs, *χ* ^*2*^ *(3*, *N* = 132) = 42.5, p = .00001. Disclosure rates were 11% (Triple P), 57% (NFP), 73% (MST) and 63% (IY). Post hoc analyses showed that disclosure rate for Triple P differed from the disclosure rate of NFP (*χ* ^*2*^ *(1*, *N* = 93) = 16.7, p = .00005), of MST (*χ* ^*2*^ *(1*, *N* = 101) = 34.7, p = .00001) and of IY (χ^*2*^ *(1*, *N* = 95) = 21.7, p = .00001). No other difference was statistically significant.

We contacted editors in connection with n = 92 articles. In the majority of publications (81/92, 86%) the editors were contacted because there was no CoI disclosure. In five cases the journal was contacted because a CoI disclosure stated that the authors actively reported no conflict of interests. Editors were also contacted in connection with eight papers in which the information provided was ambiguous or incomplete. In four of these cases the financial disclosure reported ‘no financial conflict’ while the more detailed statement allowed the reader to infer that the company pays royalties to the authors. In other cases the statement reported that the program is disseminated commercially, but it is impossible for the reader to understand the link with the authors’ personal financial interests.

In 26 cases no correction was made. Reasons why no correction was made varied: In half of the publications (n = 16) the editor reported that the journal had no CoI disclosure policy at the time of the publication. For three papers the journal consulted with the authors and reported that we had wrongly assumed that the article required a CoI disclosure. The reason was that the articles discussed a generic intervention strategy rather than an element of the intervention with which the authors had a CoI. In one case an existing CoI disclosure was considered to be satisfactory. In five cases the editors responded but were unable or unwilling to examine our request because, for example, the documents pertaining to the submission were no longer available, a different editor had been responsible for the review process at the time, or the publication of a CoI in a different journal was deemed sufficient. In three cases no response could be obtained despite three reminders.

Overall, the majority of contacts with a journal (71%, n = 65) led to an erratum, corrigendum, or addendum. For sixteen of these articles, published in nine different journals, the editors reported failures by the journal in the processing of the submitted disclosure: In fourteen cases a correct CoI disclosure had been submitted with the manuscript, but the disclosure had inadvertently been omitted. In one case the authors had correctly disclosed their conflicts of interests, and the additional statement ‘the authors declare no conflict of interests’ had been included in error.

In the remaining 49 cases the journal editors contacted the authors to request a clarification. In every case the authors responded and submitted a new or revised conflict of interest statement. This included six of the seven cases where the initial CoI disclosure actively stated that the authors declared no conflict of interests. The likelihood that a request to a journal resulted in an erratum varied between the programs, *χ* ^*2*^ *(3*, *N* = 91) = 18.7, *p* = .00031. 80% of all Triple P publications resulted in an erratum. In contrast, only one of the six FNP papers where we had contacted the journal led to an erratum or corrigendum.

### Variables associated with CoI disclosure

We conducted secondary analyses on five variables that may be associated with the likelihood of a full CoI disclosure among those that require a disclosure. The percentage of articles with a CoI disclosure increased slightly from 28% to 36% between 2008–11 and 2012–14. This difference was not statistically significant, *χ* ^*2*^ (1, *N* = 132) = 1.54, *p* = .21. Articles with the program developer as the first author had a higher disclosure rate than others (42% versus 28%) but again this difference was not significant, *χ* ^*2*^ (1, *N* = 132) = 2.02, *p* = .15. Articles published in journals that are members of COPE were significantly less likely to have a CoI disclosure than articles published in other journals (23% versus 50%), *χ* ^*2*^ (1, *N* = 132) = 10.72, *p* = .001. If a journal had a explicit disclosure policy the probability of disclosure was 40% as opposed to 16% in journals that had no such policy, *χ* ^*2*^ (1, *N* = 132) = 9.00, *p* = .003. Finally, results suggested that the likelihood of a CoI disclosure was more than twice as high if the article had been published in a medical sciences journal than in the other journals (57% versus 27%), *χ* ^*2*^ (1, *N* = 132) = 7.27, *p* = .008.

We also examined variation in the probability that our request resulted in an erratum or corrigendum among those publications for which we had contacted the editors and that potentially required a CoI (i.e. excluding categories E2 and E3, Table 1). Between the two periods (2008–11 and 2012–14) we found a small and non-significant increase in the proportion of errata from 70% to 76%, *χ* ^*2*^ (1, *N* = 88) = 0.70, *p* = .40. The chances that editors would publish an erratum were also higher if the program developer was not the first author (75%) than if the program developer was the first author (63%), but the difference was not significant, *χ* ^*2*^ (1, *N* = 88) = 2.20, *p* = .14. Whether a journal was a member of COPE or not did not affect the probability of an erratum (74% versus 68%, *χ* ^2^ (1, *N* = 88) = 0.04, *p* = .70. Journals that had a published CoI policy were much more likely to respond with an erratum (90%) as compared to those who did not have such a policy (49%), *χ* ^*2*^ (1, *N* = 88) = 21.03, *p* = 0.00001. Finally, medical journals were more likely to publish an erratum as a response to our request (90%) than non-medical journals (70%), but the difference was not significant, *χ* ^*2*^ (1, *N* = 89) = 1.52, *p* = .22.

## Discussion

The proportion of articles in peer-reviewed journals that fully disclose a personal or organisational financial CoI of the program developer was 33% among four internationally disseminated psychosocial interventions with a strong parenting support or training component. The average rate of disclosure found for these psychosocial interventions was lower than rates found in recent similar studies of pharmaceutical trials. A study on CoI disclosures among physicians found that 45.5% of publications by physicians who had obtained payments by pharmaceutical companies reported a financial CoI.[29] A study of 100 drug trial reports among Danish physicians found that almost half reported some financial CoI.[30]

However, we also found significant variation in the disclosure rates between the interventions, ranging from 11% to 70%. The reasons for this variation between the programs are not clear. The responses by the journal editors shed some light on where the responsibility for missing or incomplete CoI disclosures may lie. Specifically, 26% of all announced errata were found to result from a handling error on the part of the journal, whereby a submitted CoI disclosure had not been processed appropriately. This includes disclosures that were not published at all as well as disclosures where sections were erroneously inserted. It is difficult to assess this figure as we are not aware of any other study that has collected comparable data. However, the proportion seems high. It suggests that improved processing of disclosures at the editorial level as well as authors’ attention to completeness of the article proofs could contribute to increased transparency. At the same time, 74% of all errata (n = 46) are linked to publications where the authors had either not followed the journals’ guidance for CoI disclosure or submitted a disclosure that the editors considered incomplete in the light of our request. However, for the program with the lowest disclosure rate (Triple P) we found both more cases that had been mishandled by the editorial office and more cases where the authors were requested to submit a new or corrected CoI disclosure.

Recent studies have reported some improvement in levels of transparency about CoI as a result of the growing adoption and implementation of disclosure mechanisms, primarily in the medical sciences. [31] [32] In this study we found a slight and non-significant increase in disclosure rates since 2008. This may indicate some partial success of the efforts by COPE and other bodies that promote transparency in research. However, the findings also suggest that much remains to be done. Further analyses by subgroups suggest that more systematic controls can help further ameliorate the situation. In particular, we found that disclosure rates were significantly higher in journals with a published CoI disclosure policy and in journals in the medical sciences, where standards for CoI disclosure have been raised significantly over the past 10 years, amongst others through the introduction of the ICMJE conflict of interests disclosure form.[33] Journals that were COPE members had lower disclosure rates than journals that were not. A likely explanation for this surprising finding is that many non-members of COPE included in this study are journals published either by the APA or the American Medical Association (AMA). Journals published by these professional organizations may have promoted high standards of CoI disclosure somewhat earlier than was the case in many journals that are COPE members.

When contacted about missing or contradictory CoI statements, journal editors were generally responsive to our requests and reacted broadly in line with COPE guidance: In only 8% of all contacts we were unable to obtain a final response or the editor was unable to deal with our request.

### Additional effects

The n = 65 errata that were published as a result of our requests can be interpreted as an intervention effect. Additionally, the present study had several positive effects that are not easily quantified. One journal with no CoI policy prior to the intervention introduced such a policy as a result of the intervention. Another journal reported that it had made major changes to its CoI disclosure policy and the internal processing mechanisms as a result of our intervention. A further journal endorsed advice about possible ways to minimize the risk of non-disclosure in the future. Finally, the developer of one of the examined interventions publicly acknowledged our role in changing the way conflicts of interests are managed across the program.[34]

### Strengths

This is the first study that has systematically examined CoI disclosure for commercially disseminated psychosocial interventions. It is also the first study that examines editor compliance with COPE guidance about proper procedures, when problems are drawn to their attention. The methodological approach of contacting the journal editors provided valuable information about the responsiveness of journals to reader requests. The study also sheds some light on where the responsibility for missing CoI statements lies.

### Limitations

The observational part of the present study was limited to a convenience sample of four commercially disseminated psychosocial interventions with a strong parenting component and where there was prior published evidence of financial conflicting interests. It is not known to what extent these findings can be generalized to the wider population of psychosocial interventions. Also, no information could be obtained about why authors failed to submit CoI disclosures.

## Conclusions

Systematic reviews and meta-analyses consistently show a positive association between reported effect sizes and a CoI of the study authors.[3, 35, 36] Therefore practitioners, researchers and policy makers in public health increasingly expect transparency about conflicts of interests in academic research. Transparency about CoI in itself does not necessarily improve the quality of research, and researchers with a CoI should not be presumed to conduct less valid scholarship.[37] But transparency is a necessary component for readers to assess the study findings and their context. This study suggests that consumers of research on psychosocial interventions cannot expect that CoI disclosures are adequate and complete at present.

This is due, in part, to the absence of CoI disclosure guidelines in some scientific journals. This gap can be closed by encouraging all journals to adopt CoI disclosure policies that are widely published. Standardized and comprehensive CoI forms are an additional strategy to support compliance with published policies.[38, 39] Also, editors of nine journals reported handling errors of a submitted disclosure. Additional measures are therefore needed to improve the handling of disclosures at the level of editorial offices. Failsafe systems for CoI submission such as the ICMJE uniform disclosure formed at the point of paper submission could help minimize missing and contradictory CoI disclosure.[40]

However, the substantial variability in disclosure rates suggests that much responsibility seems to lie with the authors. The lack of transparency about authors’ reporting of COI has implications for systematic reviews in the field of psychosocial evaluations. The high rate of undisclosed COI found in the present study suggests that research syntheses relying on published information risk underestimating the extent of COI, and hence cannot reliably assess the possible association between COI and reported intervention effects. So far only one study has examined the issue in the field of psycho-social interventions.[41] It found a substantial association between an indicator that measured COI independently of the actual disclosure and the reported effect sizes.

Several measures may help to improve author compliance with standards of CoI disclosure. Universities may more actively provide support for compliance with guidance about managing CoI. To avoid inadvertent breaches of CoI guidance authors and co-author should carefully check the material at all stages of the publication process for completeness.

Finally, the present findings show that the mechanisms developed by COPE for rectifying missing CoI disclosures are generally effective, if readers document these breaches and contact editors. However, it is unknown how often readers contact editors outside the context of this study, and whether readers of the articles access the errata. Most journal editors were responsive to our requests to examine missing CoI statements. In every case where the journal editors followed our request and contacted the authors of the paper, to their credit the authors complied and submitted a correction which led to an ‘erratum’. Second, new developments such as the CONSORT extension for interventions in public health and related disciplines will likely contribute to enhance standard of reporting in the field of psychosocial interventions.[42]

Requirements about the disclosure of CoI have been in place for many years in most journals. This study examined disclosure of financial interests in situations where developers of commercially disseminated psychosocial interventions have a personal or organizational CoI. It found that disclosure rates are low and that disclosure rates vary between different interventions. As more commercial psychosocial programs appear on the market it is important that systems for effective transparency are implemented to ensure that research consumers and clinical commissioning bodies are aware of potential research biases. The field of biomedical research has optimized such systems throughout the last decade, yet our findings suggest that closer attention must be paid to these issues in psychosocial interventions.

## Supporting Information

S1 Appendix Operational Definitions of the Coding Scheme. (DOCX)

S1 Data Dataset: Articles Retrieved for Inclusion in the Study. (DOCX)
