# Supplementary material for: Disclosure of Financial Conflicts of Interests in Interventions to Improve Child Psychosocial Health: A Cross-Sectional Study
Source: PLoS One. 2015 Nov 25;10(11):e0142803. doi: 10.1371/journal.pone.0142803 (PMC4659631; doi:10.1371/journal.pone.0142803)
Supplement: S1 Appendix — (DOCX) [file pone.0142803.s001.docx]

**Appendix 1 Operational Definitions of the Coding Scheme**

| Row | Characteristic | Coding Rule |
| --- | --- | --- |
| B | Included in Analysis | Publication co-authored by the program developer, relating to the respective program, and published in an English language peer-reviewed academic journal between Jan 2008 and July 2014. |
| C | COI fully disclosed, editor not contacted | The CoI disclosure has no contradictory information (e.g. “no financial conflict of interest”) and allows the reader to infer the following information:  TP: Developer obtains royalties and research income is derived from the program dissemination.  NFP: Developer salary is partly funded by the program dissemination and research is supported by the program dissemination.  MST: Developer is co-owner of the for-profit disseminating company.  IY: Developer is co-owner of the for-profit disseminating company. |
| D1 | CoI disclosure missing | No information regarding CoI is presented in the publication. |
| D2 | "No conflict of interest" statement | The article reports “No conflict of interest” without any additional or qualifying information. |
| D3 | Ambiguous or incomplete disclosure | The article includes a Col statement that does not allow the reader to infer the information summarized in category C, or the CoI disclosure has a statement of “No financial conflict of interest” in conjunction with additional information that allows the reader to infer the existence of such an interest. |
| E1 | No disclosure policy | The editor reports that at the time of the publication the journal did not have a policy requiring authors to disclose the CoI, and that therefore no erratum will be considered. |
| E2 | Not program paper | The editor reports that the publication does not relate to a copyrighted element of the psycho-social intervention or any other program-specific treatment and that therefore a CoI is not necessary. |
| E3 | CoI considered sufficient | The editor considers the published CoI sufficiently informative. |
| E4 | Unwilling/unable to examine | The editor does not consider an erratum because he/she is unable to examine the issue, or refers us to sources outside the journal for information about the CoI situation. |
| E5 | No response | Within a period of 30 days no response is received by the editor. |
| F1 | Journal mishandling | The editor announces the publication of an erratum/corrigendum and reports that a correct CoI disclosure had been submitted with the manuscript but that a processing error led to either no publication or the publication of a contradictory CoI disclosure. |
| F2 | Authors submit corrected or new CoI | The editor contacts the author(s), agrees that an CoI disclosure would have been adequate published, and announces the publication of an erratum/corrigendum |

Note: “Row” labels refer to the labels used in table 2.
