# Supplementary material for: Disclosure of Financial Conflicts of Interests in Interventions to Improve Child Psychosocial Health: A Cross-Sectional Study
Source: PLoS One. 2015 Nov 25;10(11):e0142803. doi: 10.1371/journal.pone.0142803 (PMC4659631; doi:10.1371/journal.pone.0142803)
Supplement: S1 Data — (DOCX) [file pone.0142803.s002.docx]

**Dataset: Articles Retrieved for Inclusion in the Study**

Note:

The following list includes all 176 articles identified as relevant for the study because they were co-authored by the primary program developers and published in English between January 2008 and August 2014. The codes at the beginning of each article reflect the coding by the study authors on the basis of a reading of the article and any CoI disclosure published with the article.

Codes:

0 Paper does not relate to the commercially disseminated programme.

% Paper could not be retrieved.

+ CoI statement published and considered adequate.

* Identified as requiring clarification, COI missing or incomplete.

**Incredible Years**

+Beauchaine, T. P; Gatzke-Kopp, L., Neuhaus, E., Chipman, J., Reid, M. J. & Webster-Stratton, C. (2013). Sympathetic- and Parasympathetic-Linked Cardiac Function and Prediction of Externalizing Behavior, Emotion Regulation, and Prosocial Behavior Among Preschoolers Treated for ADHD. Journal of Consulting and Clinical Psychology, 81(3): 481–493

0Borden, L. A., Herman, K.C., Stormont, M., Goel, N., Darney, D., Reinke, W.M. & Webster-Stratton, C. (2014). Latent Profile Analysis of Observed Parenting Behaviors in a Clinic Sample. Journal of Abnormal Child Psychology, 42(5): 731-742

+Herman, Keith C., Borden, L.A., Reinke, W. M. & Webster-Stratton, C.H. (2011). The Impact of the Incredible Years Parent, Child, and Teacher Training Programs on Children’s Co-Occurring Internalizing Symptoms. School Psychology Quarterly, 26(3): 189-201

*Hurlburt, M.S., Nguyen, K., Reid, J., Webster-Stratton, C.H., Zhang J. (2013). Efficacy of the Incredible Years group parent program with families in Head Start who self-reported a history of child maltreatment. Child Abuse & Neglect, 37(8): 531– 543

%Kim, E., Choe, H. S. & Webster-Stratton, C.H. (2010). Korean Immigrant Parents' Evaluation of the Delivery of a Parenting Program for Cultural and Linguistic Appropriateness and Usefulness. Family & Community Health, 33(4): 262-274

0Mascendaro, P.M., Herman, K.C. & Webster-Stratton, C. (2012). Parent Discrepancies in Ratings of Young Children’s Co-Occurring Internalizing Symptoms. School Psychology Quarterly, 27(3): 134–143

+Presnall, N.; Webster-Stratton, C.H. & Constantino, J.N. (2014). Parent Training: Equivalent Improvement in Externalizing Behavior for Children With and Without Familial Risk. Journal of the American Academy of Child & Adolescent Psychiatry, 53(8): 879-887.e2

*Reinke, Wendy ; Stormont, Melissa; Webster-stratton, Carolyn; Newcomer, Lori & Herman, Keith (2012). The Incredible Years Teacher Classroom Management Program: Using Coaching to Support Generalization to Real-World Classroom Settings. Psychology in the Schools, 49(5): 416-428

*Taylor, T.K., Webster-Stratton C, Feil EG, Broadbent B, Widdop CS, Severson HH. (2008). Computer‐Based Intervention with Coaching: An Example Using the Incredible Years Program. Cognitive Behaviour Therapy, 37(4): 233-246

+Trillingsgaard, T., Trillingsgaard A.& Webster-Stratton C. (2014). Assessing the Effectiveness of the ‘Incredible Years Parent Training’ to Parents of Young Children with ADHD Symptoms – A Preliminary Report. Scandinavian Journal of Psychology, 55(6), 538-545.

+Webster-Stratto, C.H., Reid, M..J. & Stoolmiller, M. (2008). Preventing conduct problems and improving school readiness: evaluation of the Incredible Years Teacher and Child Training Programs in high-risk schools. Journal of Child Psychology and Psychiatry, 49(5): 471–488

+Webster-Stratton, C. H. ; Reid, M. J. &Marsenich, L. (2014). Improving Therapist Fidelity During Implementation of Evidence-Based Practices: Incredible Years Program. Psychiatric Services, 65 (6): 789-795

+Webster-Stratton, C.H. ; Reid, M.J. & Beauchaine, T. P. (2013). One-Year Follow-Up of Combined Parent and Child Intervention for Young Children with ADHD. Journal of Clinical Child & Adolescent Psychology, 42(2): 251–261

+Webster-Stratton, C.H. , Reid, M.J. & Beauchaine, T. (2011). Combining Parent and Child Training for Young Children with ADHD. Journal of Clinical Child & Adolescent Psychology, 40(2): 191-203

+Webster-Stratton, C.H. & Herman, Keith C. (2008). The Impact of Parent Behavior-Management Training on Child Depressive Symptoms. Journal of Counseling Psychology, 55(4): 473–484

*Webster-Stratton, C.H. & Herman, Keith C. (2010). Disseminating Incredible Years Series early-intervention programs: Integrating and sustaining services between school and home. Psychology in the Schools, 47(1): 36-54

*Webster-Stratton, C.H. & Reid, M. J. (2008). Adapting the Incredible Years child dinosaur social, emotional, and problemsolving intervention to address comorbid diagnoses. Journal of Children's Services, 3(3): 17-30

+Webster-Stratton, C.H., Rinaldi, J. & Reid,M.J. (2011). Long-Term Outcomes of Incredible Years Parenting Program: Predictors of Adolescent Adjustment. Child and Adolescent Mental Health, 15(1): 38–46

*Webster-Stratton, C.H.; Gaspar, M. F. & Seabra-Santos, M.J. (2012). Incredible Years® Parent, Teachers and Children’s Series: Transportability to Portugal of Early Intervention Programs for Preventing Conduct Problems and Promoting Social and Emotional Competence. Psychosocial Intervention, 21(2): 157-169

**Multisystemic Therapy**

0Ashcraft , R.G.P., Foster , S.L. , Lowery, A.E., Henggeler, S.W., Chapman, J.E. & Rowland, M.D. (2011). Measuring Practitioner Attitudes Toward Evidence- Based Treatments: A Validation Study, Journal of Child & Adolescent Substance Abuse, 20(2): 166-183

0Chapman, J.E., Sheidow, A.J. , Henggeler, S.W. , Halliday-Boykins, C.A. & Cunningham, P.B. (2008). Developing a Measure of Therapist Adherence to Contingency Management: An Application of the Many-Facet Rasch Model. Journal of Child & Adolescent Substance Abuse, 17(3): 47-68

0Clingempeel W.G., Britt S.C. & Henggeler S.W. (2008). Beyond Treatment Effects: Comorbid Psychopathologies and Long-Term Outcomes Among Substance-Abusing Delinquents. American Journal of Orthopsychiatry, 78(1): 29–36

*Foster SL, Warner SE, McCoy DM, Cunningham PB, Henggeler SW & Tiffany S. Barr (2009). Therapist Behavior as a Predictor of Black and White Caregiver Responsiveness in Multisystemic Therapy. Journal of Family Psychology, 23(5): 626–635

+Halliday-Boykins CA, Schaeffer CM, Henggeler SW, Chapman JE, Cunningham PB, Randall J, Shapiro SB (2010). Predicting Nonresponse to Juvenile Drug Court Interventions. Journal of Substance Abuse Treatment, 39(2010): 318–328

0Henggeler, S.W. & Marlow,e D.V. (2010). Introduction to Special Issue on Juvenile Drug Treatment Courts. Drug Court Review, 7(1): 1-9

+Henggeler, S.W. (2011). Efficacy Studies to Large-Scale Transport: The Development and Validation of Multisystemic Therapy Programs. The Annual Review of Clinical Psychology, 7: 351–81

+Henggeler, S.W. (2012). Multisystemic Therapy: Clinical Foundations and Research Outcomes. Psychosocial Intervention, 21(2): 181-193

*Henggeler, S.W. (2011). Treating Serious Emotional and Behavioural Problems Using Multisystemic Therapy. Australian and New Zealand journal of family therapy, 31(2): 149-164

+Henggeler, S.W. & Schoenwald, S (2011). Evidence-Based Interventions for Juvenile Offenders and Juvenile Justice Policies that Support Them. Social Policy Report, 25(1): 01-020

+Henggeler, S.W. & Sheidow AJ (2012). Empirically Supported Family-Based Treatments for Conduct Disorder and Delinquency in Adolescents. Journal of Marital and Family Therapy, 38(1): 30–58

*Henggeler, S.W., Chapman JE, Ph.D., Rowland MD, Sheidow AJ, Cunningham, PB (2013). Evaluating Training Methods for Transporting Contingency Management to Therapists. Journal of Substance Abuse Treatment, 45: 466–474

*Henggeler, S.W., Chapman JE, Rowland MD, Halliday-Boykins CA, Randall J, Shackelford J, and Schoenwald SJ (2008). Statewide Adoption and Initial Implementation of Contingency Management for Substance-Abusing Adolescents. Journal of Consulting and Clinical Psychology, 76(4): 556–567

+Henggeler, S.W., Letourneau EJ, Chapman JE, Borduin CM, Schewe PA, McCart MR (2009). Mediators of Change for Multisystemic Therapy With Juvenile Sexual Offenders. Journal of Consulting and Clinical Psychology, 77(3): 451–462

*Henggeler, S.W., McCart MR, Cunningham PB, Chapman JE (2012). Enhancing the Effectiveness of Juvenile Drug Courts by Integrating Evidence-Based Practices. Journal of Consulting and Clinical Psychology, 80(2): 264–275

+Henggeler, S.W., Sheidow, A.J., Cunningham, P.B., Donohue, B.C., Ford, J.D. (2008). Promoting the Implementation of an Evidence-Based Intervention for Adolescent Marijuana Abuse in Community Settings: Testing the Use of Intensive Quality Assurance. Journal of Clinical Child & Adolescent Psychology, 37(3): 682-689

0Holth, P., Torsheim, T., Sheidow, A.J., Ogden, T. & Henggeler, S.W. (2011). Intensive Quality Assurance of Therapist Adherence to Behavioral Interventions for Adolescent Substance Use Problems. Journal of Child & Adolescent Substance Abuse, 20(4): 289-313

+Letourneau EJ, Henggeler SW, Borduin CM, Schewe PA, McCart MR, Chapman JE, Saldana L. (2009). Multisystemic Therapy for Juvenile Sexual Offenders: 1-Year Results From a Randomized Effectiveness Trial. Journal of Family Psychology, 23(1): 89–102

+Letourneau EJ, Henggeler SW, McCart MR, Borduin CM, Schewe PA, Armstrong KS (2013). Two-Year Follow-Up of a Randomized Effectiveness Trial Evaluating MST for Juveniles Who Sexually Offend. Journal of Family Psychology, 27(6): 978–985

+McCart MR,Henggeler SW, Chapman JE, Cunningham PB, (2012). System-Level Effects of Integrating a Promising Treatment into Juvenile Drug Courts. Journal of Substance Abuse Treatment, 43: 231–243

*McCollister KE, French MT, Sheidow AJ, Henggeler SW, Halliday-Boykins CA, (2009). Estimating the Differential Costs of Criminal Activity for Juvenile Drug Court Participants: Challenges and Recommendations. Journal of Behavioral Health Services & Research, 36(1): 111-126

"+Rowland MD, Chapman JE, Henggeler SW (2008). Sibling Outcomes from a Randomized Trial of Evidence-Based Treatments with Substance Abusing Juvenile Offenders. Journal of Child & Adolescent Substance Abuse, 17(3): 11-26

+Saldana L & Henggeler, S.W. (2008). Improving Outcomes and Transporting Evidence-Based Treatments for Youth and Families with Serious Clinical Problems. Journal of Child & Adolescent Substance Abuse, 17(3): 1-10

0Schaeffer, CM, Henggele SWr, Ford JD, Mann, M, Chang RC, Chapman JE (2014). RCT of a promising vocational/employment program for high-risk juvenile offenders. Journal of Substance Abuse Treatment, 46: 134–143

*Schaeffer, CM, Henggeler SW, Chapman JE, Halliday-Boykins CA, Cunningham PB, Randall J, and Shapiro SB (2010). Mechanisms of Effectiveness in Juvenile Drug Court: Altering Risk Processes Associated with Delinquency and Substance Abuse. Drug Court Review, 7(1): 57-94

"+Schaeffer, CM, Saldana L, Rowland MD, Henggeler SW & Swenson CC (2008). New Initiatives in Improving Youth and Family Outcomes by Importing Evidence-Based Practices. Journal of Child & Adolescent Substance Abuse, 17(3): 27-45"

+Schaeffer, CM, Swenson CC, Tuerk EH, Henggeler SW (2013). Comprehensive treatment for co-occurring child maltreatment and parental substance abuse: Outcomes from a 24-month pilot study of the MST-Building Stronger Families program. Child Abuse & Neglect, 37: 596– 607

*Schoenwald S.K., Heiblum N, Saldana L, Henggeler SW (2008). The International Implementation of Multisystemic Therapy. Evaluation & the Health Professions, 31(2): 211-225

*Sheidow, A.J. & Henggeler SW (2008). Multisystemic Therapy for Alcohol and Other Drug Abuse in Delinquent Adolescents. Alcoholism Treatment Quarterly, 26(1-2): 125-145

+Sheidow, A.J., Jayawardhana J, Bradford WD, Henggeler SW & Shapiro SB (2012). Money Matters: Cost-Effectiveness of Juvenile Drug Court with and without Evidence-Based Treatments,. Journal of Child & Adolescent Substance Abuse, 21(1): 69-90

0Sheidow, A.J., Donohue, B.C., Hill, H.H., Henggeler, S.W. & Ford, J.D. (2008). Development of an Audiotape Review System for Supporting Adherence to an Evidence-Based Treatment. Professional Psychology: Research and Practice, 39(5): 553–560

+Swenson CC, Schaeffer CM, Henggeler SW, Faldowski R, Mayhew AM (2010). Multisystemic Therapy for Child Abuse and Neglect: A Randomized Effectiveness Trial. Journal of Family Psychology, 24(4): 497–507

*Tuerk EH, McCart MR, Henggeler SW (2012). Collaboration in Family Therapy. Journal of clinical psychology: In session, 68(2): 168–178

**Nurse-Family Partnership**

0 Olds, D.L. (2009). In Support of Disciplined Passion. Journal of Experimental Criminology, 5(2): 201-214

* Olds, D.L. (2008). Preventing Child Maltreatment and Crime with Prenatal and Infancy Support of Parents: The Nurse‐Family Partnership. Journal of Scandinavian Studies in Criminology and Crime Prevention, 9(S1): 2-24

* Olds, D.L. (2012). Improving the Life Chances of Vulnerable Children and Families with Prenatal and Infancy Support of Parents: The Nurse-Family Partnership. Psychosocial Intervention, 21(2): 129-143

+Bentley M.J., Lin H., Fernandez, T.V., Lee, M., Yrigollen, C.M., Pakstis, A.J., Katsovich, L., Olds, D.L., Grigorenko, E.L., Leckman, J.F. (2013). Gene variants associated with antisocial behaviour: a latent variable approach. Journal of Child Psychology and Psychiatry, 54(10): 1074–1085

0Colleen P. Crittenden; Neil W. Boris; Janet C. Rice; Catherine A. Taylor & David L. Olds (2009). The Role of Mental Health Factors, Behavioral Factors, and Past Experiences in the Prediction of Rapid Repeat Pregnancy in Adolescence. Journal of Adolescent Health, 44(1): 25-32

*Eckenrode, J.; Campa, M.; Luckey, D.; Henderson Jr, C.; Cole, R.; Kitzman, H.; Anson, E.; Sidora-Arcoleo, K.; Powers, J. & Olds, D. (2010). Long-term Effects of Prenatal and Infancy Nurse Home Visitation on the Life Course of Youths 19-Year Follow-up of a Randomized Trial. Archives of Pediatrics and Adolescent Medicine (Arch Pediatr Adolesc Med), 164(1): 9-15

*Hicks, D., Larson, C., Nelson, C., Olds, D.L. & Johnston, E. (2008). The Influence of Collaboration on Program Outcomes The Colorado Nurse–Family Partnership. Evaluation review, 32(5): 453-477

+Ingoldsby EM, Baca P, McClatchey MW, Luckey DW, Ramsey MO, Loch JM, Lewis J, Blackaby TS, Petrini MB, Smith BJ, McHale M, Perhacs M, Olds DL. (2013). Quasi-Experimental Pilot Study of Intervention to Increase Participant Retention and Completed Home Visits in the Nurse–Family Partnership. Prevention Science, 14(6): 525-534

*Jack, S.M., Ford-Gilboe, M., Wathen, C.N., Davidov, D.M., McNaughton, D.B., Coben, J.H., Olds, D.L., Macmillan, H.L.; NFP IPV Research Team. (2012). Development of a nurse home visitation intervention for intimate partner violence. BMC Health Services Research, 12(50): 1-14

+Kitzman, H.; Olds, D.; Cole, R.; Hanks, C.; Anson, E.; Arcoleo,K.; Luckey D.; Knudtson, M.; Henderson Jr, & Holmberg, J. (2010). Enduring Effects of Prenatal and Infancy Home Visiting by Nurses on Children Follow-up of a Randomized Trial Among Children at Age 12 Years. Archives of Pediatrics and Adolescent Medicine (Arch Pediatr Adolesc Med), 164(5): 412-418

+O'Brien, RA, Moritz P, Luckey DW, McClatchey MW, Ingoldsby EM, Olds DL. (2012). Mixed Methods Analysis of Participant Attrition in the Nurse-Family Partnership. Prevention Science, 13(3): 219-228

+Olds, D.; Donelan-McCall, N.; O'Brien, R.; MacMillan, H.; Jack, S.; Jenkins, T.; Dunlap III, W.; O'Fallon, M.; Yost, E.; Thorland, B.; Pinto, F.; Gasbarro, M.; Baca, P.; Melnick, A. & Beeber, L. (2013). Improving the Nurse-Family Partnership in Community Practice. Pediatrics, 132(2): 109-118

+Olds, D.; Holmberg, J.; Donelan-McCall, N.; Luckey, D.; Knudtson, M.; Robinson, j. (2014). Effects of Home Visits by Paraprofessionals and by Nurses on Children: Follow-up of a Randomized Trial at Ages 6 and 9 Years. JAMA Pediatrics, 168(2): 114-121

+Olds, D.; Kitzman, H.; Cole, R.; Hanks, C.; Arcoleo, K.; Anson, E.; Luckey, D.; Knudtson, M.; Henderson Jr, C.; Bondy, J. & Stevenson, A. (2010). Enduring Effects of Prenatal and Infancy Home Visiting by Nurses on Maternal Life Course and Government Spending Follow-up of a Randomized Trial Among Children at Age 12 Years. Archives of Pediatrics and Adolescent Medicine (Arch Pediatr Adolesc Med), 164(5): 419-424

+Olds, D.L., Kitzman, H., Knudtson, M.D,. Anson, E., Smith, J. A. & Cole, R. (2014). Effect of Home Visiting by Nurses on Maternal and Child Mortality Results of a 2-Decade Follow-up of a Randomized Clinical Trial. JAMA Pediatrics, 168(9): 800-806

*Sidora-Arcoleo, K.; Anson, E.; Lorber, M.; Cole, R. Olds, D. & Kitzman, H. (2010). Differential Effects of a Nurse Home-Visiting Intervention on Physically Aggressive Behavior in Children. Journal of Pediatric Nursing, 25(1): 35–45

0Welsh, B. C. ; Sullivan, C. J. & Olds, D. L. (2010). When Early Crime Prevention Goes to Scale: A New Look at the Evidence. Prevention Science, 11(2): 115-125

*Zielinski, D.S., Eckenrode, J., Olds, D.L.. (2009). Nurse home visitation and the prevention of child maltreatment: Impact on the timing of official reports. Development and Psychopathology, 21(1): 441–453

Triple P

*Adamson, M., Morawska, A. & Sanders, M.R. (2013). Childhood Feeding Difficulties: A Randomized Controlled Trial of a Group-Based Parenting Intervention. Journal of Developmental & Behavioral Pediatrics, 34(5): 293-302

*Bodenmann, G., Cina, A., Ledermann, T. & Sanders, M. R. (2008). The efficacy of Positive Parenting Program (Triple P) in improving parenting and child behavior: A comparison with two other treatment conditions. Behaviour Research and Therapy, 46: 411-427

*Boyle, C.L., Sanders, M.R., Lutzker, J.R., Prinz, R.J., Shapiro, C. & Whitaker, D.J. (2010). An Analysis of Training, Generalization, and Maintenance Effects of Primary Care Triple P for Parents of Preschool-Aged Children with Disruptive Behavior. Child Psychiatry & Human Development, 41(1): 114-131

0Calam, R., Jones, S., Sanders, M.R., Dempsey, R., & Sandhani, V (2012). Parenting and the emotional and behavioural adjustment of young children in families with a parent with bipolar disorder. Behavioural and Cognitive Psychotherapy, 40(4): 425-437

*Calam, R., Sanders, M.R., Miller, C., Sadhnani, V., Carmont, S. (2008). Can technology and the media help reduce dysfunctional parenting and increase engagement with preventative parenting interventions? Child Maltreatment, 13(4):347-61

%Chand, N., Farruggia, S., Dittman, C., Wai Chu, J.T. & Sanders, M.R. (2013). Promoting positive youth development through a brief parenting intervention program. Youth Studies Australia, 32(1): 29-36

*Chu, J., Farruggia, S., & Sanders, M.R., & Ralph, A. (2012). Towards a public health approach to parenting programs for parents of adolescents. Journal of Public Health, 34(s1): i41 – i47

*Clarke, S.A., Calam, R., Morawska, A. & Sanders, M.R. (2013). Developing web-based Triple P ‘Positive Parenting Programme’ for families of children with asthma. Child : care, health and development, 40(4): 492-497

%Dittman, C.K., & Sanders, M.R. (2009). The Triple P Positive Parenting programme and early childhood education. The First Years (Nga Tau Tuatahi), 11(2): 18-24

*Dittman, C.K., Farruggia, S.P., Palmer, M.L., Sanders, M.R. & Keown, L.J. (2014). Predicting Success in an Online Parenting Intervention: The Role of Child, Parent, and Family Factors. Journal of Family Psychology, 28(2): 236–243

0Dittman, C.K., Keown, L.J., Sanders, M.R., Rose, D., Farruggia, S.P., & Sofronoff, K. (2011). An epidemiological examination of parenting and family correlates of emotional problems in young children. American Journal of Orthopsychiatry, 81(3): 358-368

*Doherty, F.M., Calam, R. & Sanders, M.R. (2013). Positive Parenting Program (Triple P) for families of adolescents with Type 1 diabetes: a randomized controlled trial of self-directed Teen Triple P. Journal of pediatric psychology, 38(8): 846-858

0Evans, T., Whittingham, K., Sanders, M., Colditz, P. & Boyd, R.N. (2014). Are parenting interventions effective in improving the relationship between mothers and their preterm infants? Infant Behavior and Development, 37(2): 131-154

*Ferrari, A., Whittingham, K., Boyd, R., Sanders, M.R., & Colditz, P. (2011). Prem Triple P a new parenting intervention for parents of infants born very preterm: Acceptability and barriers. Infant Behavior and Development, 34: 602-609

*Foster, E.M., Prinz, R.J., Sanders, M.R., & Shapiro, C.J. (2008). The costs of a public health infrastructure for delivering parenting and family support. Children and Youth Services Review, 30(5): 493-501

*Frank, T.J., Keown, L.J., Dittman, C.K. & Sanders, M.R. (2014). Using Father Preference Data to Increase Father Engagement in Evidence-Based Parenting Programs. Journal of Child and Family Studies, 24(4), 937-947

*Fujiwara, T., Kato, N., & Sanders, M.R. (2011). Effectiveness of Group Positive Parenting Program (Triple P) in changing child behavior, parenting style, and parental adjustment: An intervention study in Japan. Journal of Child and Family Studies, 20: 804-813

0Haslam, D., Filus, A., Morawska, A., Sanders M.R. & Fletcher, R. (2014). The Work–Family Conflict Scale (WAFCS): Development and Initial Validation of a Self-report Measure of Work–Family Conflict for Use with Parents. Child Psychiatry & Human Development: 1-12

*Haslam, D.M., Sanders,M.R. & Sofronoff, K. (2013). Reducing Work and Family Conflict in Teachers: A Randomised Controlled Trial of Workplace Triple P. School mental health, 5(2): 70-82

0Healy, K., Sanders, M.R. & Iyer, A. (2013). Parenting Practices, Children’s Peer Relationships and Being Bullied at School. Journal of Child and Family Studies: 1-14

*Healy, K.L & Sanders, M.R. (2014). Randomized Controlled Trial of a Family Intervention for Children Bullied by Peers. Behavior Therapy, 45(6): 760-777

*Joachim, S., Sanders, M.R., & Turner, K.M.T. (2010). Reducing Preschoolers' Disruptive Behaviour in Public with a Brief Parent Discussion Group. Child Psychiatry & Human Development, 41: 47-60

*Jones, S., Calam, R., Sanders,M., Diggle,P.J., Dempsey, R. & Sadhnani, V. (2014). A Pilot Web Based Positive Parenting Intervention to Help Bipolar Parents to Improve Perceived Parenting Skills and Child Outcomes. Behavioural and Cognitive Psychotherapy, 42(3): 283-296

+Kirby, J.N. & Sanders, M.R. (2013). Using a Behavioural Family Intervention to Produce a Three-Generational Benefit on Family Outcomes: A Case Report. Behaviour Change, 30: 249-261

*Kirby, J.N. & Sanders, M.R. (2014). A randomized controlled trial evaluating a parenting program designed specifically for grandparents. Behaviour Research and Therapy, 52: 35-44

*Kirby, J.N. & Sanders, M.R. (2013). The Acceptability of Parenting Strategies for Grandparents Providing Care to Their Grandchildren. Prevention Science: 1-11

*Kirby, J.N. & Sanders, M.R. (2012). Using consumer input to tailor evidence-based parenting interventions to the needs of grandparents. Journal of Child and Family Studies, 21 (4): 626- 636

*Leung, C., Fan, A. & Sanders, M.R. (2013). The effectiveness of a Group Triple P with Chinese parents who have a child with developmental disabilities: A randomized controlled trial. Research in Developmental Disabilities, 34(3): 976–984

"%Leung, C., Sanders, M.R., Fung, B. & Kirby, J. (2014). The effectiveness of the Grandparent Triple P program with Chinese families: A randomized controlled trial. Journal of Family Studies, 20(2), 104-117.

*Love, S.M., Sanders, M.R., Metzler, C.W., Prinz, R.J. & Kast, E.Z. (2013). Enhancing Accessibility and Engagement in Evidence-Based Parenting Programs to Reduce Maltreatment: Conversations With Vulnerable Parents. Journal of public child welfare, 7(1): 20–38

*Matsumoto, Y., Sofronoff, K. & Sanders, M.R. (2010). Investigation of the Effectiveness and Social Validity of the Triple P Positive Parenting Program in Japanese Society. Journal of Family Psychology, 24(1): 87-91

*Matsumoto, Y., Sofronoff, K., & Sanders, M.R (2008). Socio-ecological predictor model of parental intention to participate in Triple P-Positive Parenting Program. Journal of Child and Family Studies, 18(3): 274-283

*Mazzucchelli T.G. & Sanders, M.R. (2014). Parenting From the Outside-In: A Paradigm Shift in Parent Training? Behaviour Change, 31 (2): 102–109

*Mazzucchelli T.G. & Sanders, M.R. (2011). Preventing behavioural and emotional problems in children who have a developmental disability: A public health approach. Research in Developmental Disabilities, 32: 2148-2156

*Mazzucchelli T.G. & Sanders, M.R. (2010). Facilitating Practitioner Flexibility within Evidence Based Practice: Lessons from a system of parenting support. Clinical Psychology: Science & Practice, 17: 238-252

0Mejia, A., Calam, R. & Sanders, M.R. (2012). A review of parenting programs in developing countries: Opportunities and challenges for preventing emotional and behavioral difficulties in children. Clinical Child and Family Psychology Review, 15: 163-175

+Mejia, A., Calam,R. & Sanders, M.R. (2014). Examining Delivery Preferences and Cultural Relevance of an Evidence-Based Parenting Program in a Low-Resource Setting of Central America: Approaching Parents as Consumers. Journal of Child and Family Studies: 1-12

*Metzler, C., Sanders, M.R., Rusby, J., & Crowley, R. (2012). Using Consumer Preference Information to Increase the Reach and Impact of Media-Based Parenting Interventions in a Public Health Approach to Parenting Support. Behavior Therapy, 43 (2): 257-70

0Morawska, A., & Sanders, M.R (2008). Parenting gifted and talented children: What are the key child behaviour and parenting issues? Australian and New Zealand Journal of Psychiatry, 42: 819-827

*Morawska, A., & Sanders, M.R. (2009). An evaluation of a behavioural parenting intervention for parents of gifted children. Behaviour Research and Therapy, 47: 463-470

*Morawska, A., & Sanders, M.R. (2009). Parenting gifted and talented children: Conceptual and empirical foundations. Gifted Child Quarterly, 53(3): 163-173

0Morawska, A., Haslam, D., Milne, D., & Sanders, M.R. (2011). Evaluation of a brief parenting discussion group for parents of young children. Journal of Developmental & Behavioral Pediatrics, 32(2): 136-145

*Morawska, A., Ramadewi, M.D., & Sanders, M.R. (2014). Using epidemiological survey data to examine factors influencing participation in parent-training programs. Journal of Early Childhood Research, 1476718X14536952.

*Morawska, A., Sanders, M.R., Goadby, E., Headley, C., Hodge, L. McAulliffe, C., Anderson, E. (2011). Is the Triple P-Positive Parenting Program acceptable to parents from culturally diverse backgrounds? Journal of Child and Family Studies, 20: 614-622

+Morawska, A., Sanders, M.R., O’Brien, J., McAulliffe, C., Pope, S., & Anderson, E. (2012). Practitioner Perceptions of the Use of the Triple P – Positive Parenting Program with Culturally Diverse Families. Australian Journal of Primary Health, 18(4), 313-320.

+Morawska, A., Tometzki, H. & Sanders M.R. (2014). An Evaluation of the Efficacy of a Triple P-Positive Parenting Program Podcast Series. Journal of Developmental & Behavioral Pediatrics, 35(2): 128-137

0Morawska, A., Winter, L., & Sanders, M.R. (2009). Parenting knowledge and its role in the prediction of dysfunctional parenting and disruptive child behaviour. Child: Care, Health and Development, 35(2): 217-226

0Nicholson, J.M., Phillips, M., Whitton. S., Halford W. K., & Sanders M.R. (2008). Promoting healthy stepfamilies: reasons for seeking help and perceived benefits from intervention. Family Matters, 77: 48-56

0Palmer, ML.., Rose, D., Sanders, M.R., & Randle, F. (2012). Conflict between work and family among New Zealand teachers with dependent children. Teaching and Teacher Education, 28: 1049-1058.

*Palmer,M.L., Henderson, M., Sanders,M.R., Keown, L.J. & White, J. (2013). Study protocol: evaluation of a parenting and stress management programme: a randomised controlled trial of Triple P Discussion Groups and Stress Control. BMC Public Health, 13(888): 1-10

0Pennell, C., Whittingham, K., Boyd, R., Sanders, M. R., & Colditz, P. (2012). Prematurity and Parental Self-Efficacy: The Preterm Parenting & Self-Efficacy Checklist. Infant Behaviour and Development, 35: 678-688

0Pickering ,J.A. & Sanders, M.R. (2013). Enhancing Communities through the Design, Development and Dissemination of Positive Parenting Interventions. Journal of Applied Research on Children: Informing Policy for Children at Risk, 4(2): 1-16

0Pidgeon, A.M., & Sanders, M.R. (2009). Attributions, parental anger and risk of maltreatment.. International Journal of Child Health and Human Development, 2(1): 57-69

*Prinz, R.J., Sanders, M.R., Shapiro, C.J., Whitaker, D.J. & Lutzker, J.R. (2009). Population-based prevention of child maltreatment: The US Triple P system population trial. Prevention Science, 10(i): 1-12.

*Roux, G., Sofronoff, K., & Sanders, M.R. (2013). A Randomized controlled trial of Group Stepping Stones Triple P: A mixed disability trial. Family Process, 52 (3): 411–424

*Salari, R., Ralph, A. & Sanders, M.R. (2014). An Efficacy Trial: Positive Parenting Program for Parents of Teenagers. Behaviour Change, 31(1): 34-52

*Salmon, K., Dittman, C., Sanders, M.R., Burson, R. & Hammington, J (2014). Does Adding an Emotion Component Enhance the Triple P Positive Parenting Program?. Journal of Family Psychology, 28(2): 244-252

*Sanders, M.R, Calam, R., Durand, M., Liversidge, T., & Carmont S. (2008). Does self-directed and web-based support for parents enhance the effects of viewing a reality television series based on the Triple P-Positive Parenting Programme?. Journal of Child Psychology and Psychiatry, 49(9): 924-32

0Sanders, M.R. (2011). Commentary on "Strengthening Families: Parents' Voices on Discipline and Child Rearing. Journal of Family Strengths, 11(1): 19.

*Sanders, M.R. (2010). Adopting a public health approach to the delivery of evidence-based parenting intervention.. Canadian Psychology, 51(1): 17-23

*Sanders, M.R. (2008). The Triple P-Positive Parenting Program as a public health approach to strengthening parenting. Journal of Family Psychology, 22(4): 506-517

*Sanders, M.R. (2012). Development, evaluation, and multinational dissemination of the Triple P Positive Parenting Program. Annual Review of Clinical Psychology, 8: 345–379

*Sanders, M.R. & Kirby J.N. (2012). Consumer engagement and the development, evaluation and dissemination of evidence-based parenting programs. Behavior Therapy, 43 (2): 1 to 19

*Sanders, M.R. & Kirby, J.N. (2014). Surviving or Thriving: Quality Assurance Mechanisms to Promote Innovation in the Development of Evidence-Based Parenting Interventions. Prevention Science, 16(3), 421-431.

*Sanders, M.R. & Kirby, J.N. (2010). Consumer involvement and population based parenting interventions. Administration, 33(2): 33-50.

*Sanders, M.R. & Mazzucchelli, T.G. (2013). The Promotion of Self-Regulation Through Parenting Interventions. Clinical Child and Family Psychology Review, 16(1): 1-17

*Sanders, M.R. & Murphy-Brennan, M. (2010). Creating conditions for success beyond the professional training environment. Clinical Psychology: Science & Practice, 17: 31-35.

*Sanders, M.R. & Pidgeon, A.M. (2011). The role of parenting programs in the prevention of child maltreatment. Australian Psychologist, 46 (4): 199–209

*Sanders, M.R. & Prinz, R.J. (2008). Ethical and professional issues in the implementation of population-level parenting interventions. Clinical Psychology: Science & Practice, 15(2),: 130-136

*Sanders, M.R., & Prinz, R. (2008). Using mass media as a population level strategy to strengthen parenting skills. Journal of Clinical Child and Adolescent Psychology, 37(3): 609-621

*Sanders, M.R., Baker, S., & Turner, K.M.T. (2012). A randomized controlled trial evaluating the efficacy of Triple P Online with parents of children with early onset conduct problems. Behaviour Research and Therapy, 50: 675-684

*Sanders, M.R., Dittman, C.K., Farruggia, S.P. & Keown L.J. (2014). A Comparison of Online Versus Workbook Delivery of a Self-Help Positive Parenting Program. Journal of Primary Prevention, 35(3): 125-133

0Sanders, M.R., Dittman, C.K., Keown, L.J., Farruggia, S., & Rose, D. (2010). What are the parenting experiences of fathers? The use of household survey data to inform decisions about the delivery of evidence-based parenting interventions to fathers. Child Psychiatry & Human Development, 41(5),: 562-569

*Sanders, M.R., Haslam, D., Calam, R., Southwell, C., & Stallman, H.M. (2011). Designing effective interventions for working parents: A web-based survey of parents in the UK workforce.. Journal of Children’s Services, 6(3): 186-200

*Sanders, M.R., Kirby, J.N., Tellegen, C.L., Day, J.J. (2014). The Triple P-Positive Parenting Program: A systematic review and meta-analysis of a multi-level system of parenting support. Clinical psychology review, 34 (4): 337-357

0Sanders, M.R., Morawska, A., Haslam, D.M., Filus, A. & Fletcher, R. (2014). Parenting and Family Adjustment Scales (PAFAS): Validation of a Brief Parent-Report Measure for Use in Assessment of Parenting Skills and Family Relationships. Child Psychiatry & Human Development, 45 (3): 255-272

*Sanders, M.R., Prinz, R.J., & Shapiro, C.J. (2009). Predicting Uptake and Utilization of Evidence- Based Parenting Interventions with Organizational, Service-Provider and Client Variables. Administration and Policy in Mental Health and Mental Health Services Research, 36: 133- 143

*Sanders, M.R., Prior, J. & Ralph, A. (2009). An evaluation of a brief universal seminar series on positive parenting: A feasibility study. Journal of Children’s Services, 4(1): 4-20.

*Sanders, M.R., Ralph, A., Sofronoff, K., Gardiner, P., Thompson, R., Dwyer, S., & Bidwell, K. (2008). Every Family: A population approach to reducing behavioral and emotional problems in children making the transition to school. Journal of Primary Prevention, 29: 197-222

+Sanders, M.R., Stallman, H., & McHale, M. (2011). Workplace Triple P: A Controlled Evaluation of a Parenting Intervention for Working Parents. Journal of Family Psychology, 25(4),: 581- 590.

0Sanders, M.R.& Burke, K. (2014). The ‘‘Hidden’’ Technology of Effective Parent Consultation: A Guided Participation Model for Promoting Change in Families. Journal of Child and Family Studies, 23(7): 1289-1297.

+Sethi, S., Kerns, S.E.U., Sanders, M.R. & Ralph, A. (2014). The international dissemination of evidence-based parenting interventions: impact on practitioner content and process self-efficacy. International Journal of Mental Health Promotion, 16(2): 126-137

*Shapiro, C.J., Prinz, R.J. & Sanders, M.R. (2010). Population-Based Provider Engagement in Delivery of Evidence-Based Parenting Interventions: Challenges and Solutions. Journal of Primary Prevention, 31: 223-234

*Shapiro, C.J., Prinz, R.J., & Sanders, M.R. (2012). Facilitators and Barriers to Implementation of an Evidence-Based Parenting Intervention to Prevent Child Maltreatment: The Triple P Positive Parenting Program. Child Maltreatment, 17(1): 84-93

*Shapiro, C.J., Prinz, R.J., & Sanders, M.R. (2008). Population-wide parenting intervention training: Initial feasibility. Journal of Child and Family Studies, 17: 457-466

*Sofronoff, K., Jahnel, D., & Sanders, M.R. (2011). Stepping Stones Triple P seminars for parents of a child with a disability: A randomized controlled trial. Research in Developmental Disabilities, 32: 2253-2262

0Stallman, H. M., Morawska, A., & Sanders, M. R. (2009). The Parent Problem Checklist: A tool for assessing parent conflict. Australian Psychologist, 44(2): 78-85

*Stallman, H.M. & Sanders, M.R. (2014). A Randomized Controlled Trial of Family Transitions Triple P: A Group- Administered Parenting Program to Minimize the Adverse Effects of Parental Divorce on Children. Journal of Divorce & Remarriage, 55(1): 33-48

+Tellegen, C.L. & Sanders, M.R (2012). Using primary care parenting interventions to improve outcomes in children with developmental disabilities: A case report. Case Reports in Pediatrics, 150261: 1 to 5

+Tellegen, C.L. & Sanders, M.R. (2013). Stepping Stones Triple P-Positive Parenting Program for children with disability: A systematic review and meta-analysis. Research in Developmental Disabilities, 34(5): 1556–1571

"*Tellegen, C.L. & Sanders, M.R. (2014). A Randomized Controlled Trial Evaluating a Brief Parenting Program with Children with Autism Spectrum Disorders. Journal of Consulting and Clinical Psychology, *82*(6): 1193.

*Tsivos, Z.L., Calam, R., Sanders, M.R. & Wittkowski, A. (2014). A pilot randomised controlled trial to evaluate the feasibility and acceptability of the Baby Triple P Positive Parenting Programme in mothers with postnatal depression. Clinical child psychology and psychiatry, 1-23

*Turner, K.M.T., Nicholson, J., & Sanders, M.R. (2011). The role of practitioner self-efficacy, training, program and workplace factors on the implementation of an evidence-based parenting intervention in primary care. Journal of Primary Prevention, 32, 95-112

0West, F., & Sanders, M.R. (2009). The lifestyle behaviour checklist: A measure of weight-related problem behaviour in obese children. International Journal of Pediatric Obesity, 1, 41852

*West, F., Sanders, M.R., Cleghorn, G.J, & Davies, P.S.W. (2010). Randomised clinical trial of a family-based lifestyle intervention for childhood obesity involving parents as the exclusive agents of change. Behaviour Research and Therapy, 48(12), 1170-1179.

*Whittingham, K., Boyd, R.N., Sanders,M.R. & Colditz, P. (2014). Parenting and Prematurity: Understanding Parent Experience and Preferences for Support. Journal of Child and Family Studies, 23: 1050–1061

*Whittingham, K., Sanders, M., McKinlay, L. & Boyd, R.N. (2014). Interventions to Reduce Behavioral Problems in Children With Cerebral Palsy: An RCT. Pediatrics, 133(5): e1249-e1257

*Whittingham, K., Sanders,M., McKinlay,L. & Boyd, R.N. (2013). Stepping Stones Triple P and Acceptance and Commitment Therapy for Parents of Children with Cerebral Palsy: Trial Protocol. Brain Impairment, 14(2): 270–280

*Whittingham, K., Sofronoff, K., Sheffield, J., & Sanders, M.R. (2009). Do parental attributions affect treatment outcome in a parenting program? An exploration of the effects of parental attributions in an RCT of Stepping Stones Triple P for the ASD population. Research in Autism Spectrum Disorders, 3: 129-144

*Whittingham, K., Sofronoff, K., Sheffield, J., Sanders, M.R. (2009). Stepping Stones Triple P: An RCT of a parenting program with parents of a child diagnosed with an Autism Spectrum Disorder. Journal of Abnormal Child Psychology, 37: 469-480

0Whittingham, K., Wee, D., Sanders, M.R. & Boyd, R. (2013). Predictors of psychological adjustment, experienced parenting burden and chronic sorrow symptoms in parents of children with cerebral palsy. Child: Care, Health and Development, 39(3): 366-373

0Whittingham, K., Wee, D., Sanders, M.R., & Boyd, R. (2013). Sorrow, Coping and Resiliency: Parents of children with Cerebral Palsy share their experiences. Disability and Rehabilitation, 35(17): 1447-52

*Wiggins, T.L., Sofronoff, K., & Sanders, M.R. (2009). Pathways Triple P-Positive Parenting Program: Effects on parent-child relationships and child behavior problems. Family Process, 48, 517–530

0Winter, L., Morawska, A., & Sanders, M.R. (2012). The Knowledge of Effective Parenting Scale (KEPS): A tool for public health approaches to universal parenting programs. Journal of Primary Prevention, 33(2): 85-97

*Winter, L., Morawska, A., & Sanders, M.R. (2012). The effect of behavioral family intervention on knowledge of effective parenting strategies. Journal of Child and Family Studies, 21(6): 881-890
